# Transient Thermal Analysis of Concrete Box Girders: Assessing Temperature Variations in Canadian Climate Zones

**DOI:** 10.3390/s23198206

**Published:** 2023-09-30

**Authors:** Musab Nassar, Lamya Amleh

**Affiliations:** Civil Engineering Department, Toronto Metropolitan University, Toronto, ON M4B 2K3, Canada; musab.nassar@torontomu.ca

**Keywords:** concrete box girder, transient thermal analysis, effective mean temperature, different climate regions, finite element, thermal gradient, validation

## Abstract

This study examines the temperature distributions and thermal-induced responses in reinforced concrete bridge elements, focusing on the Canadian climate regions. The Canadian Highway Bridge Design Code (CHBDC) currently utilizes a fixed thermal gradient profile that does not account for regional climatic variations. Historical environmental data determines the effective maximum temperatures in the CHBDC. In order to investigate temperature behaviors and distributions, a transient finite element (FE) model is developed using recorded and calculated 3-month thermal loads data for representative cities in different climate regions. The results indicate that the predicted daily maximum effective mean temperatures and extreme daily positive vertical thermal gradients do not align. A linear correlation exists between the daily maximum effective mean temperature and the daily maximum air temperature, with a coefficient of determination (R^2^) of 0.935. The proposed effective mean temperatures obtained from the FE thermal analysis are higher than the CHBDC recommendations. New thermal gradient profiles are proposed for Canadian climate zones, consisting of two straight lines and a linear gradient at the top and bottom sections. A comparison between the proposed profiles and the CHBDC and AASHTO specifications reveals that a single fixed thermal gradient profile is inadequate to account for the variation in thermal gradients across Canadian climate regions.

## 1. Introduction

The effect of climate change on the functional and structural integrity of bridge structures across Canada is considerable [1]. Increasing climate change loads accelerate the deterioration of Canadian bridges and reduce their designed service life [2]. Highway bridges are constructed in open areas and are exposed to different environmental changes. This continued exposure to climate change significantly impacts the bridge structures because of the time-dependent thermal loads [1,3]. The variations in the thermal loads, such aas air temperature, solar radiation, and wind speed, are diurnal and seasonal. The non-uniformity in the thermal loads leads to fluctuation in the mean temperature and thermal gradient values. For unrestrained bridges, thermal movements along the bridge’s span occur due to the increase and decrease in the mean temperature. On the other hand, thermal-induced stresses are developed for the restrained bridges [3] and might exceed the stresses induced by other loads [4,5]. The variations of the lateral and vertical gradients impose stresses on some bridge components and deformations in the bridge superstructures [6]. In addition, cracking phenomena can occur for some bridges due to the temperature rise [7]. The excessive thermally induced stresses, thermal-induced cracking, and thermal deformations jeopardize the bridges’ durability and safety. In some severe cases, the thermal loads and their effects can significantly cause severe damage to the major structural components [8,9].

Global research intensively investigated temperature distributions and variations due to the criticality of thermal-induced responses on bridges. Several studies have utilized experimental, finite element (FE) methods and advanced sensor technology to investigate the effects of temperature on different bridge components [10,11,12,13,14,15,16,17]. However, inaccuracies in estimating temperature distributions can lead to significant thermal stresses, resulting in undesired structural effects such as concrete cracking [18]. For instance, significant findings include Cunren Jiang et al. [10], who simulated the temperature field of a concrete box girder using ANSYS. They found that a temperature gradient of up to 8 °C could be reached for the bottom flange of the box girder, which was not considered in the bridge structure design code [10]. Xiao Lei et al. (2020) investigated prestressed-concrete bridge temperatures and thermal gradients. It was found that the lateral gradient-induced tensile stress is significant and should be considered in the design phase [11]. Abid et al. (2013) collected hourly air temperature, hourly total global solar radiation, and hourly wind speed data for around one year to study the effect of temperature variations on the box-girder segment. Empirical formulas were developed to predict the distributions of the mean temperatures and extreme lateral and vertical thermal gradients [12]. Taysi and Abid (2015) studied the thermal properties’ impact on the concrete box-girder’s thermal field. The results showed that the vertical thermal gradient sometimes exceeded the American Association of State Highway and Transportation Officials’ AASHTO’s vertical gradient by up to 10 °C [13]. Decreasing the specific heat and thermal conductivity of the concrete material combined with increasing the solar absorptivity leads to increasing the vertical thermal gradient differences and mean temperatures. These studies, among others, highlight the importance of accurately calculating temperature distributions along bridge girders to prevent adverse concrete cracks. They also highlight the need for the development of precise vertical thermal gradients. For this reason, many investigations have aimed to accurately determine vertical thermal gradients, and different bridge codes have subsequently adopted some of these proposed thermal gradients [6,19,20,21,22,23,24,25,26,27,28].

Field measurements, facilitated by temperature sensors installed on bridges, capture the most accurate data related to temperature fluctuations due to real-world climatic changes. Such measures serve as a benchmark, verifying the validity of theoretical or numerical predictions. Enhanced by the progression in sensor technology and computational power, field assessments of thermal loads on bridges have become increasingly common across various structures. The recent advancements in sensing, data acquisition, computing, communication, and data management have notably accelerated the application of structural health monitoring (SHM) technology within bridge infrastructures [29,30,31,32,33,34]. Interpolation damage detection method (IDDM) under fluctuations in thermal gradient and temperature [35] and Automated Operational Modal Analysis (AOMA) [36] were developed for proposing a new application for bridge SHM. Multiple countries reported successful deployments and operations of SHM systems on their bridges. Notably, several SHM systems encompass a temperature monitoring subsystem [37]. These sensors continuously record temperature variations over extended periods, yielding invaluable insights into temperature distributions and enabling predictions regarding potential extreme thermal loads.

Against this backdrop, this research provides a comprehensive examination of temperature distributions and thermal gradient variations in concrete box girders exposed to the diverse climatic conditions found across Canadian regions. The consequences of neglecting these variations are significant in terms of structural safety and performance. By using finite element thermal analysis, this study introduces a data-centric approach that mimics the function of a sensor, thereby aiding proactive detection of potential thermal stresses and safety hazards. Furthermore, by highlighting how climate changes influence thermal responses, this research emphasizes the importance of accurate sensing in structural engineering, aligning advanced sensor technology with practical engineering challenges to ensure the safety and long service life of bridge infrastructures. Considering future climate changes and their impact on infrastructure systems, it is crucial to investigate the thermal loads and gradients different from those recommended by the CHBDC. The assumption of a stationary thermal gradient in Canadian climate regions and the lack of an explicit thermal gradient profile raises questions that require thorough investigation. Additionally, the effect of thermal gradients on the soffit of concrete girders, a critical section to consider, was not adequately addressed in the CHBDC. This paper aims to develop daily maximum mean temperatures and positive vertical thermal gradients for different climate regions in Canada using a validated thermal model.

## 2. Materials and Methods

This study used the following methodology to investigate thermal characteristics and temperature distributions in concrete box girder bridges across different climate regions in Canada.

1. Development and Validation of 3D FE Thermal Model: A three-dimensional finite element (FE) thermal model was developed and validated using measurements from experimental thermocouples’ temperatures and thermal loads obtained from a previous study by Abid et al. [12]. An experimental full-scale reinforced concrete box-girder segment was constructed and instrumented with different sensors to measure and record the environmental thermal loads (the air temperature, solar radiation, and wind speed) for over a year. Three sensor types, including the 108-temperature probe, the three-cup NRG#40 anemometer, and the CS3 apogee silicon pyranometer, were used to monitor the air temperature, wind speed, and global solar radiation intensity, respectively. A considerable number of type-T thermocouples (62 thermocouple sensors) were installed inside the vertical webs and the top and bottom flanges and on the exterior and interior surfaces of the flanges and the webs to measure concrete temperatures. The experimental measurements were gathered over a period exceeding one year, commencing in May 2013 and concluding in July 2014. This validated FE thermal model formed the basis for further analysis in this research [12].
2. Determination of Daily Maximum Mean Temperatures and Thermal Gradient Variations: The validated FE thermal model was utilized to determine the daily maximum mean temperatures, also known as effective temperatures, extreme positive vertical thermal gradient variations, and positive thermal gradient profiles for each representative city in the different climate regions of Canada. Emphasis was placed on the investigation of positive vertical thermal gradient variations, which are considered the most extreme gradients resulting from the combined effects of high solar radiation and temperature fluctuations.
3. Transient Thermal Models for Representative Cities: Transient thermal models were developed for each representative city, incorporating time-dependent environmental loads such as solar radiation, ambient air temperature, and average wind speed. These models facilitated a comprehensive analysis of the thermal behavior and distribution of the concrete box girder within each climate region.
4. Comparison with CHBDC Recommendations: The maximum effective temperatures for the representative cities were compared with those recommended by the CHBDC. This comparison aimed to assess any disparities and identify potential deviations from the CHBDC specifications.
5. Comparison of Proposed Thermal Gradient Variations and Profiles: The proposed thermal gradient variations and profiles specific to the Canadian climate regions were compared with the thermal gradient profiles and values specified by both CHBDC and the AASHTO. This analysis aimed to highlight any discrepancies and demonstrate the insufficiency of a single fixed thermal gradient profile in capturing the variations across different Canadian climate regions.

## 3. Data Acquisition for Thermal Model Verification and Thermal Analysis

**Figure 1:**
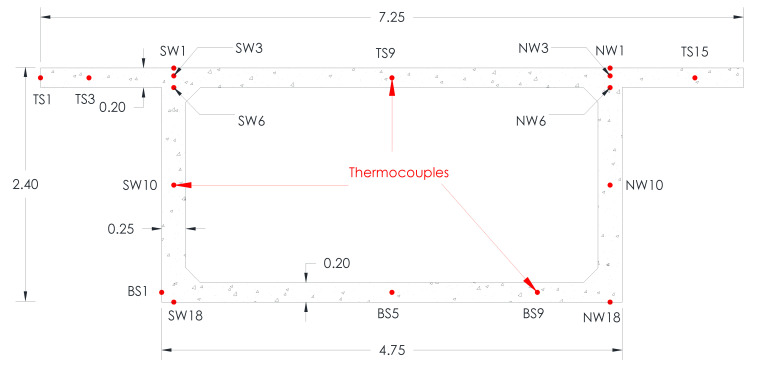
Box-girder cross-section, including the locations of the thermocouples (unit: m).

The study aimed to verify and validate the thermal model used in the analysis of concrete box-girder bridges. To achieve this, thermal load data was gathered from a previous study conducted by Abid et al. in 2016. The data included ambient air temperature, total global solar radiation, and wind speed, collected over a year, and temperature measurements obtained from strategically placed thermocouples (sensor that measures temperature) on the box-girder segment’s surfaces. The specific analysis focused on data from different days between 11 June 2014 and 14 June 2014, which provided a range of environmental conditions to evaluate the thermal behavior of the concrete box girder. In particular, the thermal loads of ambient air temperature, total global solar radiation, and wind speed were observed and recorded. Figure 1 displays the cross-section of the box girder, showing the positions of the thermocouples used for model verification. The width of the top and bottom flanges of the concrete segment are 7.25 m and 4.75, respectively. The length of the overhang is 1.25 m. The thickness of the top and bottom slabs is 0.2 m, while the thickness of the vertical webs is 0.25 m. The overall height and length of the concrete segment are 2.4 m and 2.1 m, respectively. Temperature measurements taken on 14 June 2014 were crucial for the study’s temperature verification. By utilizing the comprehensive thermal load data and thermocouple readings from Abid et al.’s previous study, the thermal model employed in this study can be verified and validated, ensuring its suitability for conducting thermal analysis on concrete box-girder bridges.

## 4. Heat Transfer Equation Theory and Thermal Boundary Conditions

The Fourier heat transfer differential equation governs the concrete bridge’s heat conduction. For the 3D thermal analysis and because the bridge is assumed not to be subjected to the internal heat source, the heat transfer differential equation can be formulated as shown in Equation (1) [38,39]. The heat generated during cement hydration affects the thermal behavior of the structure during the early age of concrete. However, the effect of hydration heat on the temperature field of concrete can be very limited after the first few days following concrete casting. Moreover, in the subsequent days and as the effect of hydration heat decreased, the thermal distribution and behavior were more dependent on the environmental thermal loads. Therefore, the hydration heat of cement can only be considered during the early age of concrete (the initial stages of concrete’s lifespan), and it can be neglected during the later stages of concrete lifespan. In this study, the hydration heat of cement was not considered in the FE model. Boundary conditions and initial conditions are required to solve the Fourier heat conduction equation.

(1) $$\rho C\frac{\partial T}{\partial t}=k(\frac{\partial^{2}T}{\partial x^{2}\;}+\frac{\partial^{2}T}{\partial y^{2}}+\frac{\partial^{2}T}{\partial z^{2}})$$

where *ρ* is the material’s destiny and c is the material’s specific heat in J/kg °C. k represents the material thermal conductivity coefficient in W/m °C, and T represents the temperature at position (x,y,z) at time t. x, y, and z represent the cartesian coordinates.

When the concrete bridge is exposed to climate change, heat energy exchange occurs between the bridge’s surfaces and the surrounding atmosphere. Three main heat transfer mechanisms occur on the box girder’s boundaries. These heat transfer mechanisms are solar radiation and solar irradiation, convection heat transfer, and heat conduction [38,39]. The boundary conditions equation that describes the thermal loads on the girder’s surfaces is expressed in Equation (2) [38].

(2) $$\;k\frac{\partial T}{\partial n}l+q=0\;$$

where l is the direction cosine (x,y,z) of the unit outward vector perpendicular to the boundary and q represents the summation of the heat fluxes exchange between the surface of the girder section and the surrounding environment in W/m^2^, which is presented in Equation (3).

(3) $${q=q}_{s}{+q}_{\mathrm{con}}{+q}_{\mathrm{re}}$$

where ${\;q}_{s}$, $q_{\mathrm{con}}$, and $q_{\mathrm{re}}$ are total solar radiation heat flux (short-wave), convective heat flux, and long-wave radiation heat flux in W/m^2^, respectively. The total solar radiation heat flux includes the beam solar radiation heat flux ${\;q}_{b}$, diffuse solar radiation heat flux ${\;q}_{d}$, and the ground reflected radiation heat flux ${\;q}_{\mathrm{gr}}$, as expressed in Equation (4).

(4) $$q_{s}{=q}_{b}{+q}_{d}{+q}_{\mathrm{gr}}$$

### 4.1. Beam Solar Radiation

The beam solar radiation (${\;I}_{b}$) reaches the Earth’s surface without being diffused. The beam solar radiation heat flux is affected by the value of the girder section’s absorptivity (α).

Calculation of the beam solar radiation (${\;I}_{b}$) on different surfaces depends on solar altitude angle (h), solar incident angle (θ), Solar Azimuth Angle (φ), declination angle (D), solar zenith angle (β), surface Azimuth Angle (ψ), atmospheric transparency coefficient (P), and other factors [40]. The Bouguer-Lambert law is usually used as a rough method to determine the ground beam solar radiation ($I_{m}$), as shown in Equation (5) [41,42].

(5) $${\;I}_{m}=P{\;I}_{0}$$

where P is the atmospheric transparency coefficient given in Equation (6) and I_0_ is the solar constant given in Equation (7) [43], which depends on the day of the year (n) calculated from January 1st as n = 1.

(6) $$P=0.9^{\frac{k_{a}t_{u}}{\sin\left(h\;+5\right)}}$$

where $k_{a}$ represents the relative atmospheric pressure, which can be calculated based on the altitude value. $t_{u}$ is the turbidity factor that depends on the atmospheric conditions, $t_{u}$ ranges between 1.8 and 3. For this research, the turbidity coefficient is assumed to be the average value (2.4). h is the solar altitude angle.

(7) $$I_{0}=1367(1+0.033\cos\frac{360n}{365})$$

The beam radiation component ($I_{b}$) striking the structure surfaces, such as the box-girder’s web and top flange, is expressed in Equation (8) [40].

(8) $$I_{b}\;=\;I_{m}\cos(\theta )$$

where θ represents the solar incidence angle between the incident sunlight and the normal surface. The solar incident angle is determined as shown in Equation (9). When the beam solar component strikes a horizontal surface, the incident angle θ and the solar zenith angle β are equal. Further details about determining the solar incident angle and other geometric factors can be found in the research conducted by Kalogirou and Peng [40,42].

(9) cos θ=cos δ cos β +sin δ sin β cos (φ–ψ)

where δ is the tilted angle, representing the angle between the surface and the horizontal plane. β is the solar zenith angle, which is the angle between the sun’s rays and the vertical direction of the Earth. β can be calculated as shown in Equation (10). φ is the solar azimuth angle, which is determined using Equation (11). ψ refers to the surface azimuth angle, the angle between the normal to the surface from true south; the angle is zero due south, westward, and eastward and is designated as positive and negative, respectively.

cos β = cos D cos H cos L + sin D sin L(10)

cos φ = sec h (cos D cos H sin L − sin D cos L(11)

D refers to the declination angle that does not depend on the location and depends on the date (n the number of days starting from the first of the year with Jan 1 as n = 1) as expressed in Equation (12). H is the hour angle, which is zero at noon. Morning hours are designated as negative, while afternoon hours are positive. L refers to the latitude angle where the concrete girder is built. h is the solar altitude angle expressed in Equation (13), which refers to the angle between the sun’s solar beam and the horizontal plane.

(12) $$D=23.45\sin\;(360\times \frac{284+n}{365})$$

Sin h = Cos β(13)

### 4.2. Diffuse Solar Radiation

Diffuse solar radiation reaches the earth’s surface after it was scattered from the beam solar beam by the atmosphere. The diffuse scattering radiation on an inclined surface ($I_{d}$) can be formulated as shown in Equation (14) [43].

(14) $$I_{d}={\;I}_{\mathrm{dh}}\frac{1+\;\cos\delta \;}{2}$$

where $I_{\mathrm{dh}}$ is expressed in Equation (15), which is the diffuse solar radiation component that strikes the horizontal surface [44].

(15) $$I_{\mathrm{dh}\;}=(0.271-0.294P)\;I_{0}\;\cos\;\beta$$

### 4.3. Ground-Reflected Radiation

The ground-reflected radiation originates from the beam and diffuses solar radiation striking the horizontal surface, which is expressed as Equation (16) [43].

(16) $$I_{\mathrm{gr}\;}=r_{g\;}\;(I_{\mathrm{bh}\;}+I_{\mathrm{dh}\;})\;\frac{1-\cos\delta \;}{2}$$

where $r_{g\;}$ represents the reflection coefficient of the ground (albedo), and for this research, $r_{g\;}$ is applied mostly as a value of 0.2 for ground reflection [11,45]. $I_{\mathrm{bh}\;}$ and $I_{\mathrm{dh}\;}$ combined is the total radiation striking the horizontal surface. 

The girder surfaces’ total heat fluxes are reduced by the girder section’s absorptivity (α), as expressed in Equation (17)

(17) $$q_{s\;}=\alpha \;(I_{b\;}+{\;I}_{d\;}+{\;I}_{\mathrm{gr}\;})$$

### 4.4. Heat Transfer Convection and Long-Wave Radiation

The convectional heat transfer between the exposed surfaces and the surrounding air can be determined by the following Equation (18).

(18) $$q_{\mathrm{con}\;}={\;h}_{c\;}\left(T_{s\;}-T_{a\;}\right)$$

where $h_{c\;}$ is the heat transfer convection coefficient of the surface in W/m^2^.k, $T_{s\;}$ represents the surface’s temperature, and $T_{a\;}$ represents the ambient air temperature.

For the concrete box girder, empirical formulas are applied to calculate the surface convection coefficient $h_{c}$ on different surfaces, and they all depend on the wind speed (u) in m/s. Equation (19) represents $h_{c\;}$ for the top flange’s surface, Equation (20) represents $h_{c}$ for the bottom surfaces, Equation (21) represents $h_{c\;}$ for the exterior vertical surfaces, and Equation (22) represents $h_{c}$ for the box-girder inside surfaces [43,46].

(19) $$h_{c}\;=\;3.83u\;+\;4.67$$

(20) $$h_{c}\;=\;3.83u\;+\;2.17$$

(21) $$h_{c}\;=\;3.83u\;+\;3.67$$

(22) $$h_{c}=\;3.5$$

$q_{\mathrm{re}}$ as shown in Equation (23), represents the long-wave radiation, which is the thermal radiosity of the exposed surfaces to the atmosphere. $q_{\mathrm{re}}$ also indicates the heat transfer between the bridge surface and the surrounding atmosphere due to thermal irradiation. $q_{\mathrm{re}}$ depends on the emissivity of the surface (ϵ) and the Stefan–Boltzmann constant (F), which is equal to 5.67 × 10^−8^ W/m^2^ k^4^.

(23) $$q_{\mathrm{re}}\;=\;\epsilon F\;(T_{s}^{4}-{\;T}_{a}^{4})$$

### 4.5. Overhang Shading

The girder webs are exposed to the shadow of the concrete deck overhang. The shadow generated on the web changes with time and differs based on the web surface’s inclination angle, the solar angle of incidence, and the top flange’s overhang length $\left(L_{c}\right)$. The overhang shadows significantly affect the magnitude of the solar radiation on the web. The beam solar radiation is neglected when the surface is shaded, and only the diffuse and reflected solar radiations are considered. The shadow length generated by the top flange ($L_{s}$) can be determined using Equation (24).

(24) $$L_{s\;}={\;L}_{c\;}\frac{\tan\;h\;}{\cos\;\left(\varphi -\;\psi \right)}$$

As explained earlier, the boundary conditions of heat exchange (q) and the heat transfer equation are closely tied to the thermal loads and thermal material properties. However, they are insufficient for conducting thermal analysis on a concrete box-girder in a straightforward manner. In order to tackle this challenge, implementing finite element simulation becomes essential for performing thermal analysis effectively.

## 5. Transient Thermal Analysis Using ANSYS

The heat transfer differential equations and boundary conditions equations, which govern the heat conduction within the concrete box-girder and the thermal loads are solved and simulated using the finite element simulation ANSYS [47]. A transient thermal module is utilized to analyze the concrete box girder’s transient temperature behavior over time using a mechanical APDL solver in ANSYS. The choice of mechanical APDL-ANSYS (2022 R2) over other finite element analysis (FEA) software packages such as ABAQUS and COMSOL was influenced by several factors. First, ANSYS provides specialized modules such as ANSYS Mechanical APDL simplifying the application of thermal boundary conditions. Second, ANSYS has a larger user base, well-established research support, and an extensive material library, making it a convenient and user-friendly option for this study. To apply the thermal boundary conditions accurately, the model is divided into four categories:

(1) The top flange’s surface,
(2) The bottom flange’s surface and the subsurface of the overhangs,
(3) The inside surfaces of the girder,
(4) The girder’s outside surfaces of the webs and front and back surfaces of the concrete girder, considering the shading effect due to overhang and sun movement.

The solar angles and their directions and the solar incident angle were calculated based on the latitude, the longitude, the date of the year, the time zone, the surface azimuth angle, and other geometric parameters using Equations (9)–(13) [46]. Hourly heat fluxes of different solar radiations, including beam, diffuse, and reflected solar radiation components as described in Equations (5)–(17), are determined and applied to the corresponding boundaries in the finite element model. The outside surfaces of the top and bottom parts of the girder, in addition to the webs, are exposed to different solar radiation components and fluxes. Direct and diffuse sky radiation (global solar radiation) that can be calculated using Equations (5)–(15) are absorbed by the top flange. The bottom surfaces are exposed to horizontal scattering and ground reflection radiation, which are calculated based on Equations (14) and (16). Direct solar radiation calculated based on solar incident angle and overhang shading, diffuse solar radiation, and ground-reflected solar radiation can be absorbed by the webs and the vertical parts. Overhang shading Equation (24) significantly affects the calculation of total solar radiation on the web since the web shadow area does not absorb direct solar energy. In this case, direct radiation should be subtracted from the total solar radiation on the shaded part of the web. The thermal boundary conditions on the front and back faces of the girder depend on the orientation of the girder, sun movement, and other factors. The vertical direct and diffuse solar radiation and the ground-reflected radiation were applied on the front and back faces of the girder while considering the shading effect due to sun movement. It should be noted that the thermal boundary conditions applied to the top and bottom slabs and the vertical webs exert a more pronounced influence on temperature distributions and thermal gradients compared to those applied to the front and back surfaces of the segment.

**Figure 2:**
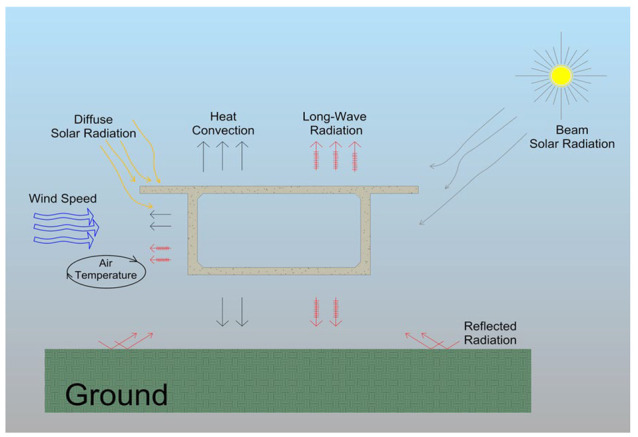
The thermal boundary conditions applied to the FE concrete box girder model.

The heat transfer coefficients shown in Equations (19)–(22) were calculated at each time step, and the air temperature was applied for the different model surfaces to govern the convection heat transfer thermal load. Long-wave radiation between surfaces shown in Equation (23) is usually applied to the thermal model using the radiosity solver and surface-to-surface correlation. In this way, the radiation calculations between surfaces are performed by calculating the view factors between surfaces and using the FE temperatures. However, for simplicity and efficiency, researchers often ignore mutual radiation (surface-to-surface radiation) [13,48,49]. To provide a visual representation of how the thermal boundary conditions are applied to the concrete girder’s thermal finite element model, a diagram illustrating the boundary environmental thermal loads is shown in Figure 2. The wind speed, ambient air temperature, and solar radiation data required for thermal model verification are obtained from the study conducted by Taysi and Abid (2016) [12], specifically recorded between 11 June 2014 and 14 June 2014.

**Figure 3:**
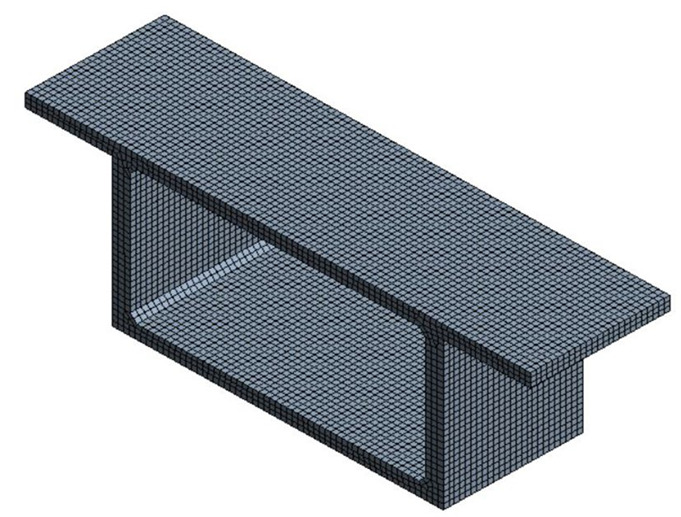
The FE mesh of the concrete box girder.

The quadratic and triangle elements were used to mesh the FE model using the sweep method, as shown in Figure 3. The element size is reduced to improve the accuracy of the transient thermal analysis. The simulated model has 8597 elements and 12,156 nodes. The number of time steps used in the transient thermal model is 192, with a time step of 0.5 h. The thermal material properties significantly affect the heat transfer and boundary conditions, as explained in Equations (1) and (2). Reducing the concrete material’s specific heat and thermal conductivity while simultaneously increasing its solar absorptivity leads to higher vertical thermal gradient variations and mean temperatures, as illustrated by Taysi and Abid (2015) [13]. This could potentially result in temperatures that exceed values recommended by the code. In this study, the thermal properties of concrete used in the FE simulation are density (2400 kg/m^3^), thermal conductivity (1.6 W/m.K), specific heat (950 J/kg.K), surface emissivity (0.85), and absorption coefficient (0.5) [13]. It should be noted that increasing the thermal conductivity and heat transfer of the concrete material leads to reduced thermal gradient variations and mean temperatures. However, conducting parametric studies to investigate the impact of these material properties on the thermal field of concrete box girders falls outside the scope of this research.

Applying an initial temperature is crucial for governing the time-dependent transient heat conduction. The initial temperature is typically chosen around midnight, as it approximates a uniform temperature distribution at that time. To minimize the initial time’s effect on the FE-predicted temperature, the initial temperature is set around midnight three days before the day of interest (14 June 2014). This approach helps to stabilize the transient thermal analysis and provide accurate temperature predictions.

## 6. Validation of ANSYS Thermal Model

**Figure 4:**
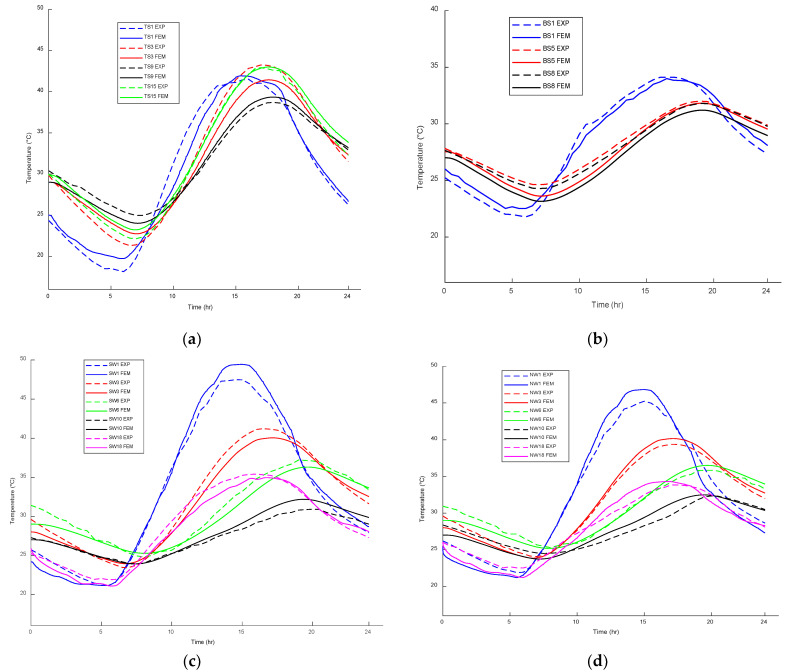
Temperature variation comparison between the proposed analytical FE and those obtained from the field temperatures for the (**a**) top surface of the slab, (**b**) bottom surface of the slab, (**c**) south surface of the web, and (**d**) north surface of the web.

**Figure 5:**
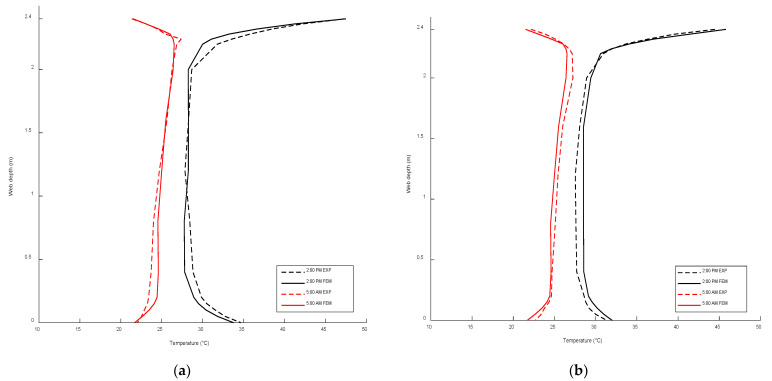
Vertical thermal gradient comparison between the proposed analytical FE and those obtained from the field temperatures along the (**a**) south web at 5:00 am and 2:00 pm and (**b**) north web at 5:00 am and 2:00 pm.

To validate the accuracy of the conducted thermal model, the field temperatures for different thermocouples shown in Figure 1 were compared with the predicted finite element temperatures for 14 June 2014. The predicted FE and experimental temperatures for different thermocouples were compared for the top and bottom slab surfaces and south and north web surfaces, as demonstrated in Figure 4, respectively. The FE and field temperatures along the web section were compared as an additional validation tool, as shown in Figure 5. The results of the comparison demonstrate a high level of agreement between the FE temperatures and the corresponding thermocouple measurements, with the highest temperature difference being approximately 2.5 °C. This indicates that the FE model accurately captures the thermal behavior of the concrete box girder under investigation. Based on these results, it can be concluded that the FE model is reliable and suitable for developing temperature distributions and thermal gradients for different climate regions across Canada. This validation ensures that the developed temperature distributions and thermal gradients can be utilized with confidence in analyzing the thermal responses of bridge structures in different climate regions of Canada.

## 7. Temperature Distributions and Variations for Different Climate Regions of Canada

**Figure 6:**
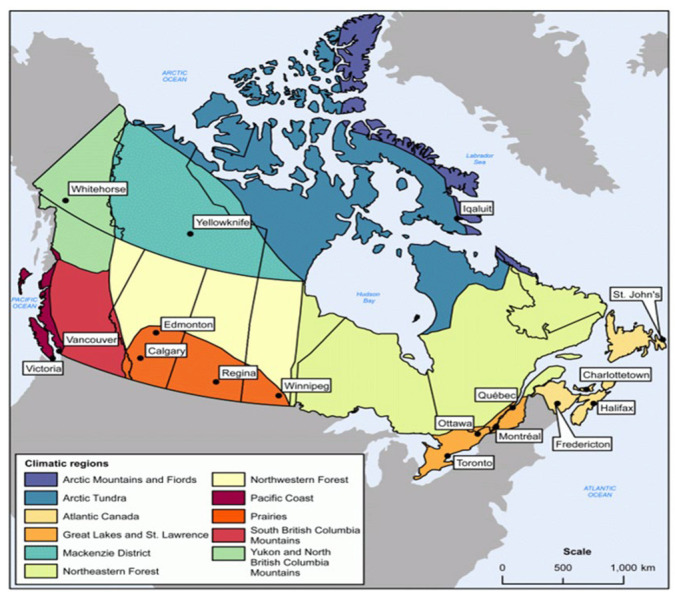
The representative cities of the Canadian climate regions [50].

Canada is characterized by a diverse range of climate regions, each with its own unique meteorological conditions that significantly impact temperature distributions and variations. However, as mentioned earlier, the CHBDC includes one positive vertical thermal gradient pattern for all Cities [26]. The Atmospheric Environment Service of Environment Canada identified 11 major climate regions in the country, as shown in Figure 6. However, the representative cities of seven climate regions shown in Figure 6 were selected to conduct the thermal analysis and to investigate the effect of climatic variations on the thermal field of the concrete box girder. These selected representative cities cover a broad spectrum of thermal loads, including air temperature and solar radiation, and diverse geographical coordinates. The thermal conditions and geographical coordinates in the excluded climate regions fall within the extensive range represented by the selected regions. Consequently, the proposed effective temperature and thermal gradient profiles can be reasonably and appropriately applied to concrete box girder bridges constructed in any Canadian climate region. To address this limitation and capture the regional variations in temperature, representative cities were carefully selected from different major climate regions. The chosen climate regions include Great Lakes and St. Lawrence (region 1), Prairies (region 2), Atlantic Canada (region 3), Pacific Coast (region 4), Yukon and North British Columbia (region 5), Mackenzie District (region 6), and Arctic Mountains and Fiords (region 7). In order to ensure comprehensive analysis and capture a wider range of solar radiation components and air temperature data, more than one city was selected to represent certain climate regions due to the geographical diversity within those regions. The selection of representative cities was based on their geographical scale, coordinates, and coverage of different climatic characteristics. The chosen cities provide a robust basis for determining the thermal gradients and temperature distributions in the respective climate regions.

The coordinates, including latitude, longitude, and altitude, of the selected representative cities for each climate region are summarized in Table 1. These coordinates are crucial inputs for the transient thermal analysis conducted in this study, enabling the accurate simulation of temperature variations and gradients across different regions of Canada. By considering multiple representative cities from various climate regions, this research aims to comprehensively understand the thermal behavior of bridge structures in Canada. The resulting temperature distributions and variations will contribute to a more accurate assessment of the thermal responses and effects on bridge components, enhancing the design and durability considerations for infrastructure in different climate regions across the country.

## 8. Thermal Loads Data for the Transient Thermal Analysis

It is important to highlight that the thermal load data used in this study serves different purposes. Initially, the experimental data obtained from Gaziantep, including ambient air temperature, global solar radiation, and wind speed, were solely utilized to verify the accuracy of the ANSYS thermal model. Once the model was validated, the focus shifted towards determining the temperature field of the concrete box girder in various climate regions of Canada using extreme thermal load data specific to the Canadian context. To capture the diverse climatic conditions across Canada, hourly environmental data such as ambient air temperature and average wind speed were obtained from Environment Canada [51]. In order to develop the daily maximum, mean temperature, and positive vertical thermal gradient pattern for different climate regions of Canada, a 3-month of data from June 1 to August 31 during the last 13 years (2010–2022) are used as thermal inputs for the transient thermal simulation. This approach was chosen to capture seasonal variability, enhance data reliability, and improve prediction accuracy. Additionally, it provides a representative climatic sample, captures inter-annual variations, and aligns with established climatological practices. This comprehensive approach aims to offer more rigorous and universally applicable insights into the thermal behaviors of concrete box girders. The maximum effective (mean) temperature is highly correlated to the maximum air temperature, typically occurring during the summer months. Therefore, the data from the summer months that witnessed the maximum hourly air temperature is selected for thermal analysis, ensuring the accurate determination of the daily maximum mean temperature. On the other hand, A different 3-month data was selected because the maximum vertical positive gradient is expected to occur for high solar radiation combined with high air temperature variations, which are expected to occur in summer [19,23]. Therefore, three summer months with the maximum difference in the daily temperatures are selected to develop the positive vertical thermal gradient. The hourly ambient air temperature and the hourly average wind speed starting from 1 June to 31 August are used to develop the daily maximum mean temperature. Solar radiation components have a significant effect on the temperature field. Beam solar radiation, diffuse solar radiation, and reflected solar radiation on the surfaces of the concrete girder are determined using Equations (5)–(16). The hourly solar radiation components are determined for all representative cities from 1 June to 31 August for both transient thermal simulations. Ambient air temperature, hourly average wind speed, and hourly solar radiation data are then used to determine the thermal boundary conditions explained in Section 4 and used as thermal inputs in the FEM simulations.

## 9. Three-Month FE Simulations

The 3-month FE simulations are conducted using the thermal loads’ data (hourly temperature, hourly average wind speed, and hourly solar heat fluxes). Two different 3-month FE simulations are conducted separately, once to develop the daily maximum mean temperature and another time to develop the maximum positive thermal gradient for each representative city of the climate regions. The same approach used for thermal validation explained in Section 5 is adopted to conduct the 3-month FE simulations with slight differences. The thermal boundary conditions are applied on the surfaces based on the calculations in Section 4 and the methodology in Section 5 using the environmental 3-month data. Transient thermal analysis with a time step of 1 h and 2208 steps is conducted to investigate the concrete box-girder’s temperatures and thermal gradients. By conducting these three-month FE simulations, the study aims to provide valuable insights into the temperature distributions and thermal gradients experienced by concrete box girders in different climate regions of Canada. This fine-grained time resolution enables a detailed investigation of temperature variations and thermal gradients, capturing the transient nature of heat transfer processes. The results obtained from these simulations will facilitate a comprehensive understanding of the thermal response of the girder under varying environmental conditions over an extended period, contributing to more informed bridge design and maintenance practices.

## 10. Daily Maximum Mean Temperatures and Daily Maximum Thermal Gradient Variations

**Figure 7:**
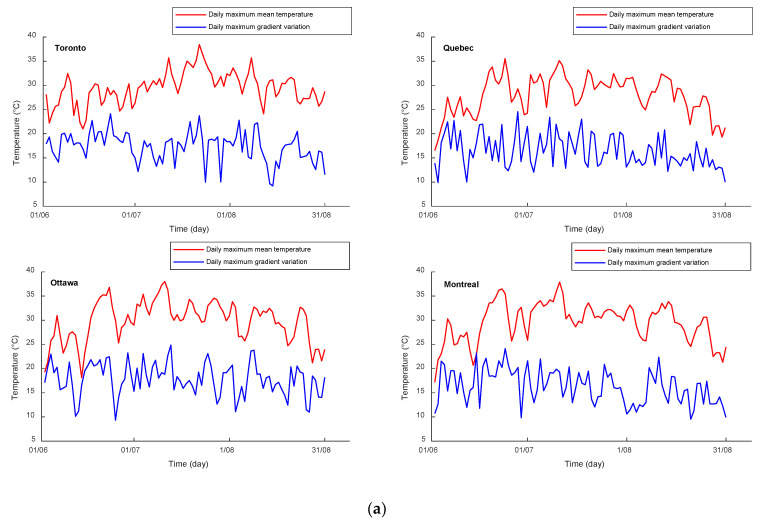

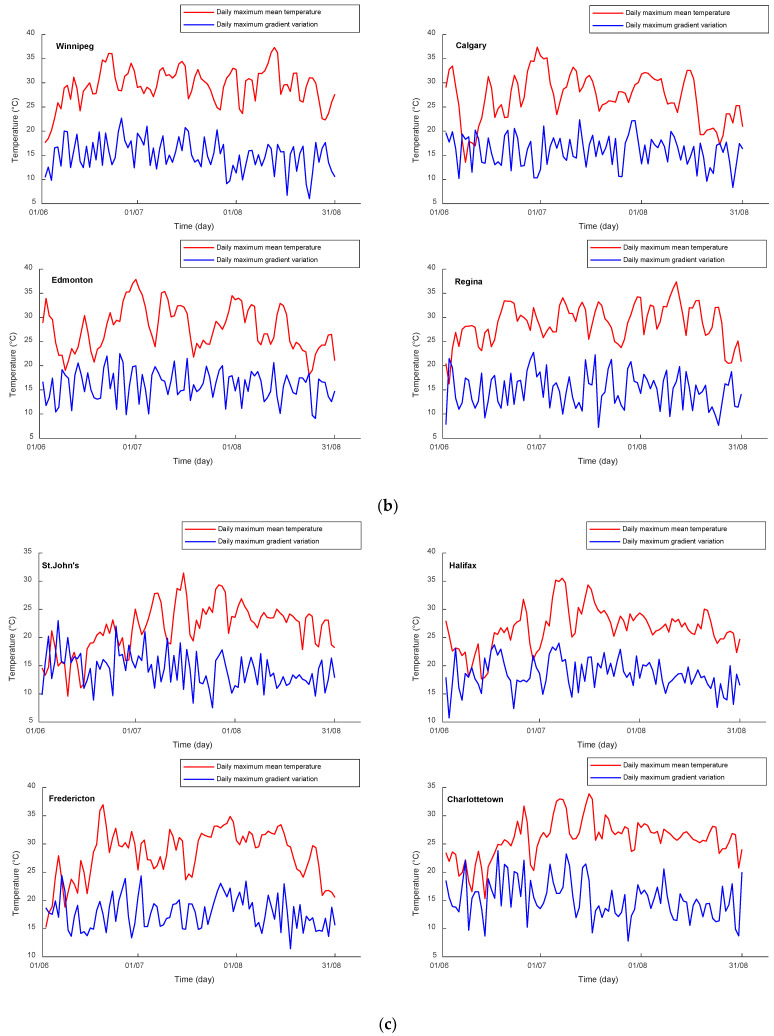

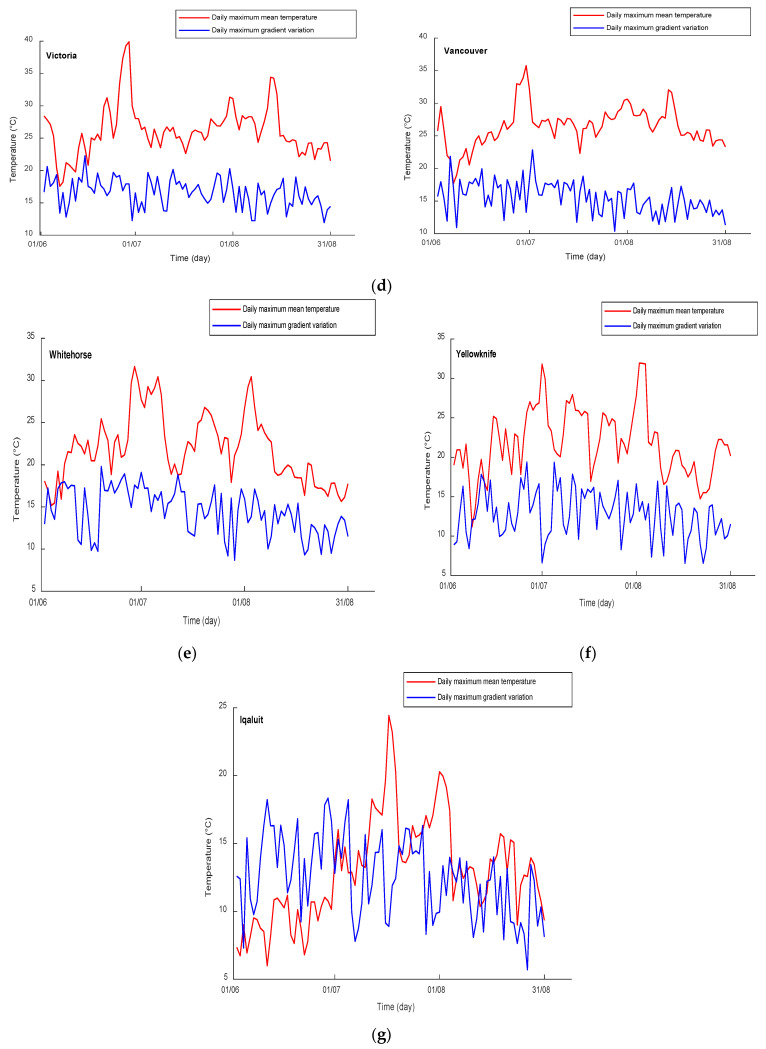
The daily maximum mean temperatures and positive gradient variations for the representative cities of 7 climate regions: (**a**) Great Lakes and St. Lawrence, (**b**) Prairies, (**c**) Atlantic Canada, (**d**) Pacific Coast, (**e**) Yukon and North British Columbia, (**f**) Mackenzie District, and (**g**) Arctic Mountains and Fiords.

The daily maximum mean temperature (DMTmax), which corresponds to the maximum effective daily temperature term based on CHBDC [26], represents the average temperature of the concrete segment and is used for expansion calculations (thermal movement). To develop DMTmax, the hourly mean temperature (MT) must be calculated. For each representative city in the climate regions, the MT is calculated for the three summer months by summing the temperatures multiplied by the surrounding area at different locations across the girder segment and dividing the sum by the total area of the girder, as shown in Equation (25). The highest value among the estimated hourly mean temperatures for each day represents the daily maximum mean temperature. In addition to DMTmax, the maximum thermal gradient variation is also analyzed. This variation refers to the temperature difference between the highest temperature in the top flange and the lowest temperature in the web during the daytime. The thermal gradient, which represents the slope of the temperature difference, is determined accordingly. Figure 7 illustrates the daily maximum mean temperatures and positive gradient variations for the representative cities in Canadian climate regions from June 1 to August 31.

**Figure 8:**
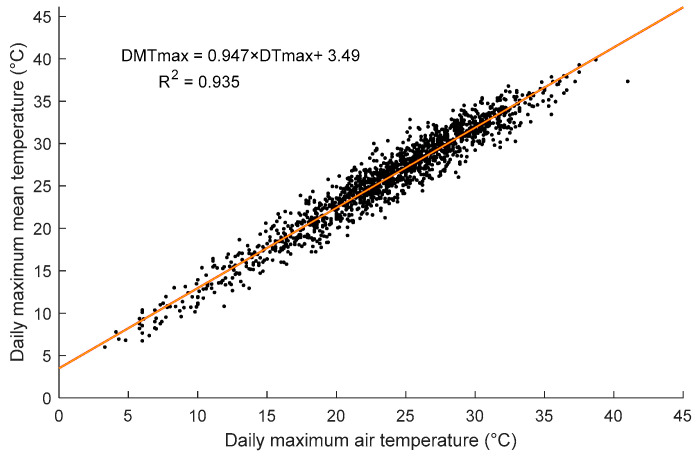
The correlation between the daily maximum mean temperature and daily maximum air temperature for the concrete girder segment.

The results presented in Figure 7 show that regardless of the representative cites, the extreme daily mean temperatures of regions 1, 2, 3, 4, 5, 6, and 7 are 38.43 °C, 37.8 °C, 36.97 °C, 39.9 °C, 31.64 °C, 31.93 °C, and 24.43 °C, respectively. The extreme value of the daily maximum mean temperature (effective temperature) for each representative city corresponds to the highest value of DTmax. This indicates that the daily maximum mean temperatures might be correlated to their corresponding daily maximum air temperatures during the three-month period. The correlation of DMTmax vs. DTmax for all representative cities for the three summer months is illustrated in Figure 8. Figure 8 shows that the DMTmax is highly correlated to DTmax with a coefficient of determination R^2^ of 0.935. The proposed formula of the DMTmax is given in Equation (26). Figure 7 shows that the extreme gradient variations for regions 1, 2, 3, and 4 ranged from 24.1 °C to 24.72 °C, 22.36 °C to 22.76 °C, 23.3 °C to 24.46 °C, and 22.58 °C to 22.85 °C, respectively. The extreme gradient variations of regions 5, 6, and 7 are 19.81 °C, 19.46 °C, and 18.34 °C, respectively.

(25) $$\mathrm{MT}\;=\frac{\sum T_{i}A_{i}}{\sum A}$$

(26) DMTmax = 0.947 × DTmax + 3.49

where Ti is the temperature at the hour step and Ai is the surrounding area of the point where the temperature is extracted from the validated FE model. A is the total cross-sectional area of the girder segment.

**Figure 9:**
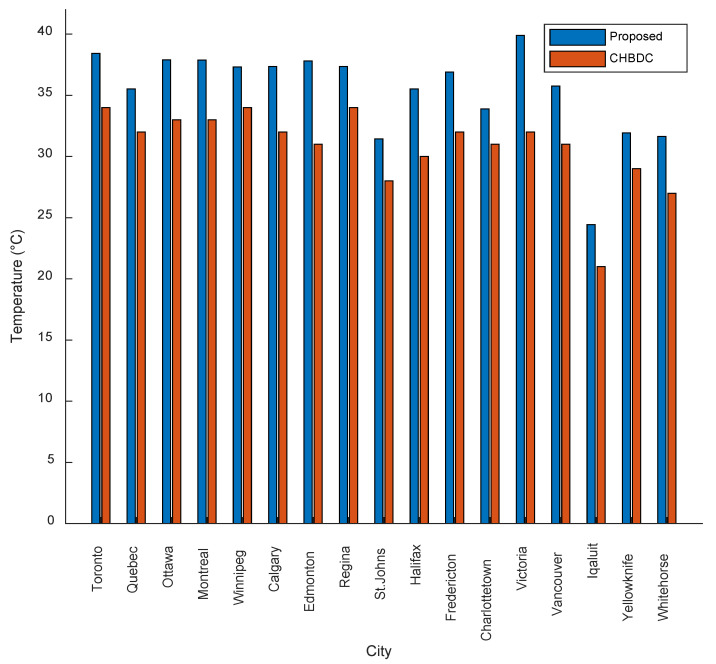
Comparison of the proposed daily maximum mean temperatures and CHBDC maximum effective daily temperatures for the representative cities of the climate regions.

To assess the validity of the proposed daily maximum mean temperatures, a comparison is made with the maximum effective daily temperatures recommended by the CHBDC, as shown in Figure 9. The results observed in Figure 9 show that the proposed daily maximum mean temperatures are consistently 3 °C more than the CHBDC maximum effective daily temperatures for all representative cities. In some cases, the difference reaches around 5 °C. This discrepancy suggests that the CHBDC underestimates the maximum effective daily temperatures used for thermal movement calculations, potentially compromising the durability and serviceability of bridge structures. As the maximum effective temperature increases, the expansion thermal movement of a bridge also increases. If this movement is restrained, it can result in unfavorable stresses, jeopardizing the bridge’s integrity and causing structural damage. Therefore, it is crucial to consider the potential impact of increasing temperatures on bridge structures and ensure that appropriate measures are taken to mitigate any potential risks to their integrity. Further research combining experimental and FE studies is recommended to obtain long-term data and explore different girder configurations. This will facilitate a more accurate determination of the maximum effective daily temperatures, contributing to enhanced bridge design and ensuring the long-term performance and safety of these structures.

## 11. Thermal Gradient Profiles for the Canadian Climate Regions

**Figure 10:**
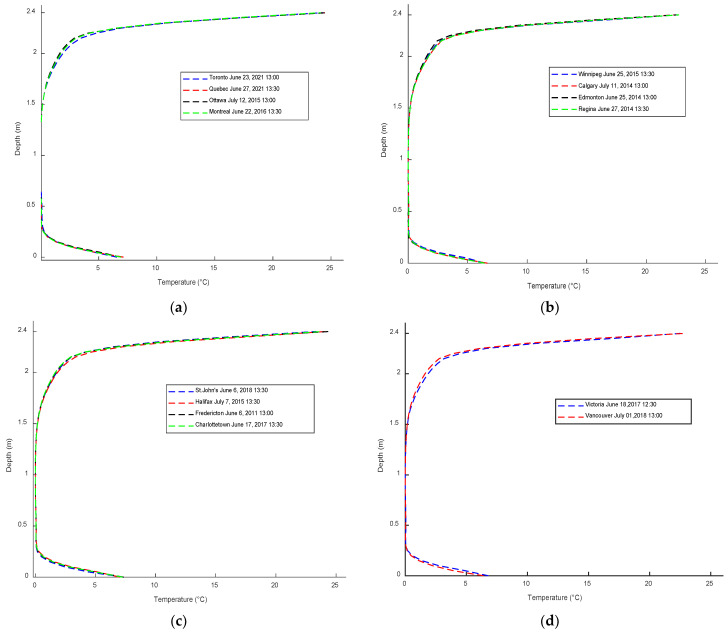

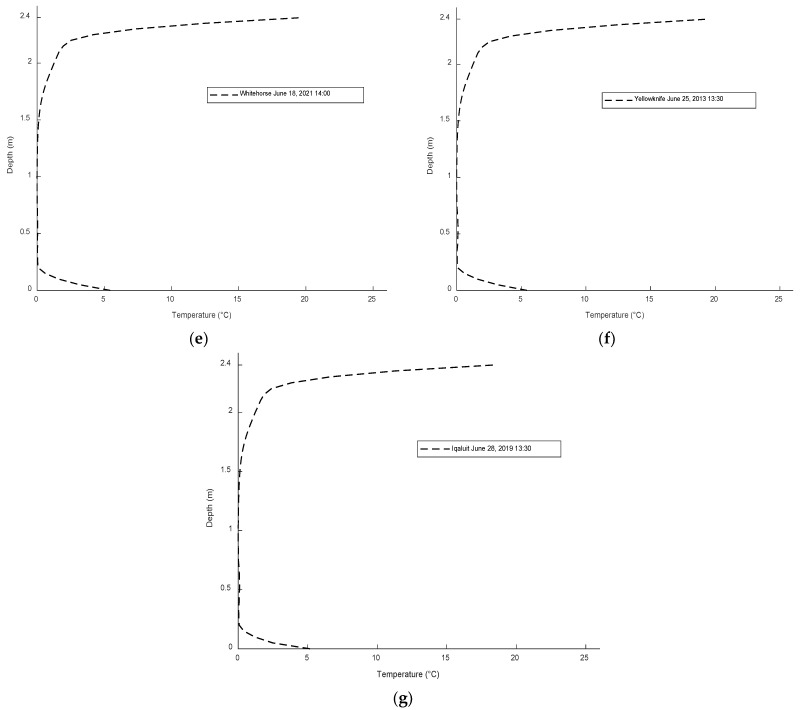
Positive vertical thermal gradient profiles for the representative cities of 7 climate regions: (**a**) Great Lakes and St. Lawrence, (**b**) Prairies, (**c**) Atlantic Canada, (**d**) Pacific Coast, (**e**) Yukon and North British Columbia, (**f**) Mackenzie District, and (**g**) Arctic Mountains and Fiords.

To determine the maximum positive vertical thermal gradient profile for each city, the gradient variation along the web is analyzed. The temperature distribution along the web depth, where the maximum gradient variation occurs, is adjusted so that the minimum temperature in the web is set to zero. This allows for the visualization of the maximum positive vertical thermal gradient profiles for the representative cities in the different climate regions, as illustrated in Figure 10. Figure 10 showcases the thermal gradient profiles for the representative cities, revealing a similar pattern among them. However, there are notable differences in the extreme thermal gradient variations observed across the climate regions. It should be noted that this study primarily focused on investigating the positive thermal gradient, and the examination of negative and lateral thermal gradients fell outside the scope of the research.

## 12. Development of the Proposed Thermal Gradient Profiles for the Canadian Climate Regions

Based on the observed thermal gradient patterns and the variations in Figure 7 and Figure 9, the thermal gradient profiles and variations of climate regions 1 and 3 are comparable and higher than the thermal gradient variations for the rest of the climate regions. This is mainly attributed to the higher solar radiation of climate regions 1 and 3 than climate regions 2, 4, 5, 6, and 7. On the other hand, climate regions 2 and 4, as well as climate regions 5, 6, and 7, exhibit similar thermal gradient variations. As a result, Canada can be divided into three climate zones: Climate Zone 1 represents climate regions 1 and 3, while Climate Zones 2 and 3 represent climate regions 2 and 4, and climate regions 5, 6, and 7, respectively. 

**Figure 11:**
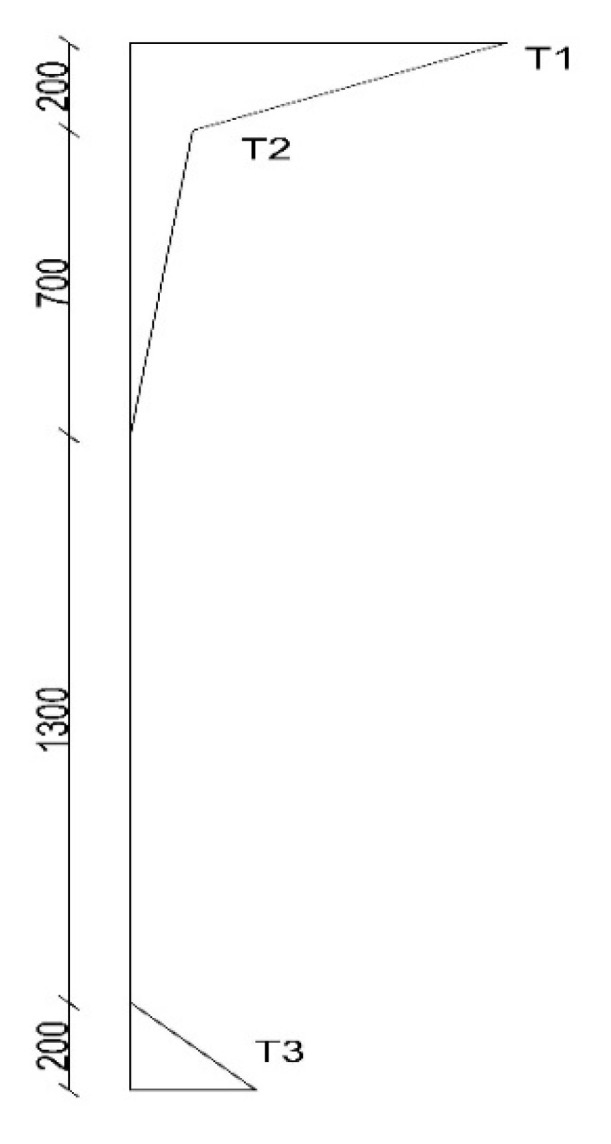
The proposed positive thermal gradient profile.

The proposed positive thermal gradient profile for each climate zone is composed of two straight-line segments (bilinear) for the top section of the girder, accompanied by an additional positive linear gradient for the girder’s bottom section, as shown in Figure 11. For the two straight-line segments, the extreme thermal gradient (T1) occurs at the deck surface, followed by the second thermal gradient (T2) at a depth of 0.2 m from the deck surface. The thermal gradient then reaches zero at a depth of 0.9 m from the top slab. For the bottom section, the positive linear gradient (T3) decreases linearly to zero at a height of 0.2 m from the girder’s bottom surface. The maximum thermal gradient variations at the top and bottom surfaces (T1 and T3) are proposed to be the highest values among the climate regions, rounded to the nearest 0.5. Table 2 provides a summary of the thermal gradients (T1, T2, and T3) for each climate zone.

**Figure 12:**
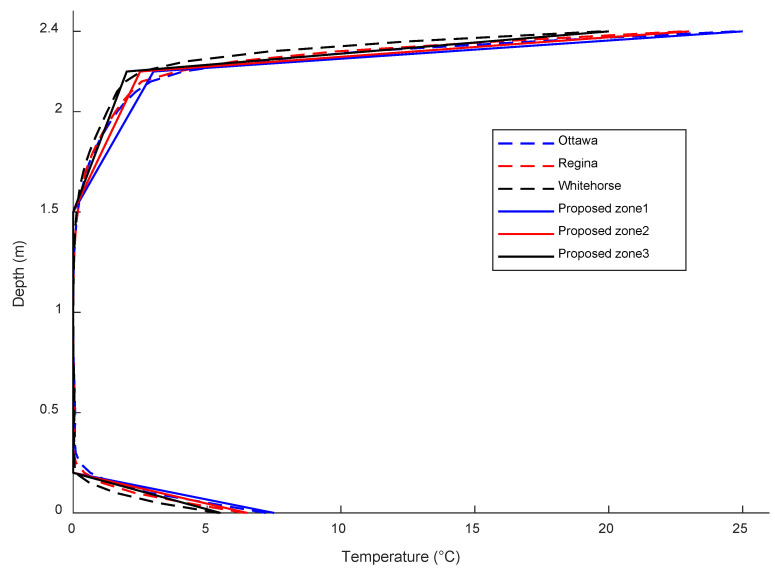
The comparison between the proposed thermal gradient profiles for the climate zones and the predicted thermal gradient profiles for the representative cities.

**Figure 13:**
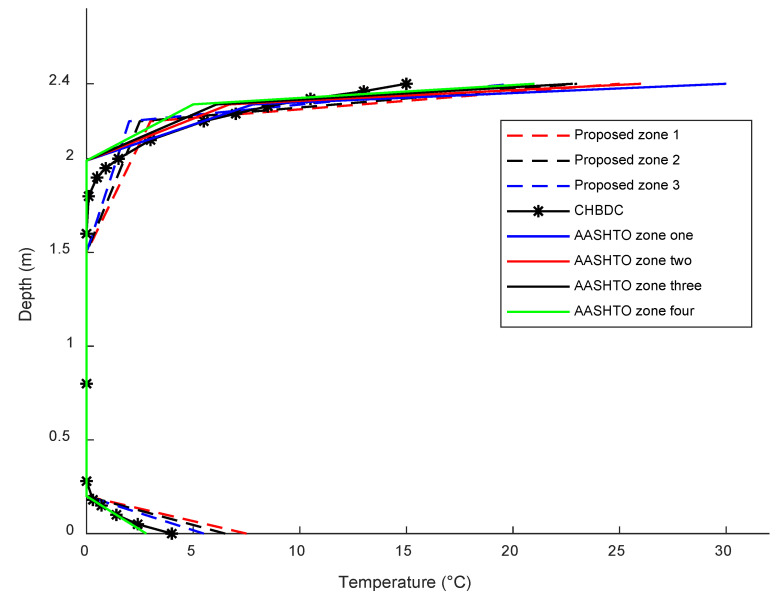
Comparison of the proposed thermal gradient profiles and CHBDC and AASHTO thermal gradient profiles.

To validate the accuracy of the proposed gradient model, the thermal gradient profiles for the climate zones are compared with the profiles of three representative cities selected from the corresponding climate regions of each climate zone, as shown in Figure 12. The comparison demonstrates a good agreement between the proposed and predicted thermal gradient profiles, further confirming the reliability of the proposed model. Furthermore, the proposed thermal gradient profiles are compared with the thermal gradient profiles recommended by the CHBDC [26] and the AASHTO [23] in Figure 13. In CHBDC, the calculation of the maximum effective temperature considers the superstructure type and requires adjustments based on its depth. Specifically, it is calculated as the maximum mean daily temperature plus a constant specific to the superstructure type. Additionally, a reduction adjustment is necessary for the maximum effective temperature calculations, which depends on the depth of the superstructure [26]. The maximum mean daily temperatures for numerous Canadian cities were initially recorded several decades ago, spanning approximately 30 years until 1970. Subsequently, these records were reasonably updated by incorporating hourly temperature observations from 76 stations across Canada for the period spanning 1959 to 2008 [26,27]. The CHBDC provides a fixed thermal gradient profile in terms of the temperature differential to account for the positive thermal gradient effect. The positive gradient variation was developed based on the depth of the structure, disregarding the differences in the regional climatic conditions. The same temperature differential profile was used for concrete and steel superstructures. However, the CHBDC commentary [27] refers to a nonlinear thermal gradient profile developed by Elbadry and Ghali [28], which is adopted in this research for comparison purposes. The thermal gradient profiles recommended by AASHTO consider different climate zones and provide varying thermal gradient values for each zone. It is evident from Figure 13 that the thermal gradient profiles proposed for the Canadian climate zones exhibit distinct patterns compared to the gradient profiles recommended by CHBDC and AASHTO codes. The CHBDC thermal gradient profile is insufficient and conservative in reflecting the proposed thermal gradient profiles for the Canadian climate zones. Additionally, the CHBDC underestimates the extreme thermal gradients at the top and bottom surfaces for all climate regions, which may lead to higher thermal-induced stresses that could potentially affect the structure’s durability. While the thermal gradient profiles recommended by AASHTO are more applicable due to considering different climate zones, the maximum proposed thermal gradient variations for each climate zone are similar to the extreme thermal gradient variations recommended by AASHTO for their respective zones. The maximum proposed thermal gradient variations for climate zone 1, climate zone 2, and climate zone 3 are approximately the same as the extreme thermal gradient variations recommended by AASHTO for zone 2, zone 3, and zone 4, respectively.

## 13. Conclusions

This study focused on the thermal analysis of concrete box girders in different climate regions of Canada. Via the utilization of finite element simulation using ANSYS, transient temperature distributions and thermal gradients were analyzed. The study considered various factors such as solar radiation, ambient air temperature, and wind speed to accurately model the thermal loads and boundary conditions. The validation of the ANSYS thermal model was successfully conducted by comparing the predicted temperatures with field measurements from thermocouples (sensor that measures temperature). The agreement between the model predictions and experimental data confirmed the reliability and accuracy of the developed thermal model.

The core of our findings highlights the pivotal role of thermal loads, which are paramount in gauging thermal stresses and subsequently inform a bridge’s service life and operational efficiency. While leaps were made in grasping bridge thermal loads via theoretical blueprints, computational analytics, and on-field measurements, our understanding still holds room for refinement, and current design guidelines cater primarily to conventional bridge configurations.

The study then proceeded to analyze the temperature distributions and variations for different climate regions in Canada. Daily maximum predicted mean temperatures are highly correlated with daily maximum collected air temperatures (R^2^ = 0.935), allowing a formula to be proposed for daily maximum mean temperature. By incorporating three months of thermal load data from June to August over multiple years, the daily maximum mean temperatures and maximum positive thermal gradient variations were determined. These findings highlighted the significant differences in temperature patterns across the climate regions and emphasized the need for region-specific thermal analysis in bridge design and analysis. Based on the observed thermal gradient patterns and variations, the study proposed thermal gradient profiles for three climate zones in Canada. These profiles accounted for the differences in solar radiation and temperature variations in each zone. The proposed profiles were compared with the thermal gradient profiles recommended by the CHBDC and the AASHTO. It was found that the proposed profiles provided a more accurate representation of the thermal conditions in Canadian climate regions and addressed the limitations and underestimations of the existing codes. The development of the proposed thermal gradient profiles has significant implications for the design and analysis of concrete box girders in Canada. By considering the specific thermal conditions of each climate zone, engineers and designers can make informed decisions regarding material selection, expansion joint placement, and structural detailing to ensure the long-term durability and integrity of bridge structures. The significant differences between proposed and CHBDC thermal values highlight the need for further research using long-term data, different girder configurations, and different thermal material properties to investigate maximum effective daily temperatures and thermal gradients accurately.

The start of SHM technology streamlined the process of automated, real-time temperature monitoring on bridges. Despite several bridges being equipped with temperature sensors, the acquired data often needs to be more utilized, in contrast to vibration and stress data, which receive extensive analysis. The impact of thermal loads on bridge performance needs to be more adequately understood. Thus, comprehensive field measurements across diverse bridges and regions should be promoted. Furthermore, the primary focus should be deriving thermal load models from field data.

Solely relying on data from temperature sensors to describe the temperature distribution across a bridge needs to be improved. Numerical models can serve as robust complements. However, there is a need for more sophisticated numerical models that factor in all essential elements to simulate temperature variations in large bridges accurately. Factors such as the intensity of solar radiation, the relationship between air temperature and wind speed, and the dynamics of heat exchange with the surrounding environment require more precise characterization. Additionally, seamlessly transitioning from heat transfer analysis to thermal stress analysis remains an area that needs to be addressed.

It is important to note that this study focused primarily on positive thermal gradients and did not investigate negative or lateral thermal gradients. Further research could explore these aspects to provide a more comprehensive understanding of thermal effects on bridge structures. In addition, the uncertainty in projected air temperature distributions may lead to higher variations in thermal gradients, necessitating more research on various concrete box girder sections to address this concern.

## Figures and Tables

**Table 1:** The coordinate system of the representative cities.

Climate Region	Representative Cities	Latitude and Longitude	Altitude (m)
	Toronto	43°40′00.000″ N 79°24′00.000″ W	166
	Ottawa	45°24′00.000″ N 75°43′00.000″ W	115
Region 1	Montreal	45°28′00.000″ N 73°30′00.000″ W	36
Great Lakes and St. Lawrence	Quebec	46°48′00.000″ N 71°13′00.000″ W	76
	Calgary	51°06′00.000″ N 114°20′00.000″ W	1099
	Edmonton	53°33′00.000″ N 113°30′00.000″ W	671
Region 2	Regina	50°25′55.000″ N 104°39′57.000″ W	578
Prairies	Winnipeg	49°55′00.000″ N 97°14′58.000″ W	239
	St. John’s	47°34′00.000″ N 52°42′00.000″ W	141
	Charlottetown	46°14′00.000″ N 63°10′00.000″ W	51
Region 3	Halifax	44°50′00.000″ N 55°03′00.000″ N	145
Atlantic Canada	Fredericton	45°52′19.670″ N 66°31′40.410″ W	25
	Victoria	47°50′00.000″ N 77°22′00.000″ W	20
Region 4			
Pacific Coast	Vancouver	49°18′00.000″ N 123°01′00.000″ W	16
Region 5	Whitehorse	60°43′00.000″ N 135°03′00.000″ W	706
Yukon and North British Columbia			
Region 6	Yellowknife	62°27′46.000″ N 114°26′25.000″ W	206
Mackenzie District			
Region 7		63°45′00.000″ N 68°33′00.000″ W	34
Arctic Mountains and Fiords	Iqaluit		

**Table 2:** The thermal gradients T1, T2, and T3 for the Canadian climate zones.

Climate Zone	T1 (°C)	T2 (°C)	T3 (°C)
Zone 1	25	3	7.5
Zone 2	23	2.5	6.5
Zone 3	20	2	5.5

## Data Availability

The authors confirm that the data supporting the findings of this study are available within the article. However, additional data can be provided upon reasonable request.
